# The Core Proteome and Pan Proteome of *Salmonella* Paratyphi A Epidemic Strains

**DOI:** 10.1371/journal.pone.0089197

**Published:** 2014-02-24

**Authors:** Li Zhang, Di Xiao, Bo Pang, Qian Zhang, Haijian Zhou, Lijuan Zhang, Jianzhong Zhang, Biao Kan

**Affiliations:** 1 State Key Laboratory for Infectious Disease Prevention and Control, National Institute for Communicable Disease Control and Prevention, Chinese Center for Disease Control and Prevention, Beijing, P. R. China; 2 Collaborative Innovation Center for Diagnosis and Treatment of Infectious Diseases, Hangzhou, P.R.China; State Key Laboratory of Pathogen and Biosecurity, Beijing Institute of Microbiology and Epidemiology, China

## Abstract

Comparative proteomics of the multiple strains within the same species can reveal the genetic variation and relationships among strains without the need to assess the genomic data. Similar to comparative genomics, core proteome and pan proteome can also be obtained within multiple strains under the same culture conditions. In this study we present the core proteome and pan proteome of four epidemic *Salmonella* Paratyphi A strains cultured under laboratory culture conditions. The proteomic information was obtained using a Two-dimensional gel electrophoresis (2-DE) technique. The expression profiles of these strains were conservative, similar to the monomorphic genome of *S.* Paratyphi A. Few strain-specific proteins were found in these strains. Interestingly, non-core proteins were found in similar categories as core proteins. However, significant fluctuations in the abundance of some core proteins were also observed, suggesting that there is elaborate regulation of core proteins in the different strains even when they are cultured in the same environment. Therefore, core proteome and pan proteome analysis of the multiple strains can demonstrate the core pathways of metabolism of the species under specific culture conditions, and further the specific responses and adaptations of the strains to the growth environment.

## Introduction

Over 2500 serotypes have been reported in *Salmonella*, and most of them result in diarrhea. Within these serotypes, *Salmonella enterica* serovar Typhi and Paratyphi, can lead to systemic infections in humans, known as typhoid and paratyphoid fever. These diseases cause epidemics in Asia, Africa and Latin America [1], [2]. Before the 1990s, *S*. Typhi was the main causative agent of enteric fever in southeast Asia and in China, but in the mid-1990s, the number of cases caused by *S.* Paratyphi A started to increase, and paratyphoid fever subsequently became the major enteric fever [3], [4], [5], [6].

The whole genomes of some *S*. Typhi and *S*. Paratyphi A strains have been sequenced [7], [8], [9], [10]. Genetically monomorphic genomes and relatively low sequence diversity were found, which may be the result of a high restriction of host adaption [11]. Multi-locus sequence typing (MLST) and pulsed-field gel electrophoresis (PFGE) [12] were used to generate phylogenetic information and obtain a population variance analysis, and for *S.* Typhi and *S*. Paratyphi A genotyping. Genomic sequencing and a single nucleotide polymorphism (SNP) analysis provided high-throughput and high-resolution genome variation methodology [13], and were applied for the epidemic analysis of *S.* Typhi strains [14], [15], [16], [17]. All of the results showed a low level of genetic variation in *S*. Paratyphi A, and a high clonality of strains involved in epidemics.

A genome comparison among different strains is used to identify the core genome and pan genome [18]. The core genome includes the core, conserved genes and surviving characteristics which keep the microorganism evolving. In contrast, the pan genome includes newly transferred genes, and demonstrates the diversity of the organism. Genome comparisons help investigators discover the divergence of the same genes between different organisms. However, a genome analysis cannot show the differences in the protein levels, which are the actual determinants of the growth and survival of the organism. Proteomic studies can illustrate the expression levels of various gene products under given culture conditions, discover the responses to different biological systems and uncover protein modifications and protein-protein interactions [19], [20]. A comparison of the proteomes of different strains can indicate their shared and unique features. Besides the shared proteins, it may also help identify newly acquired gene products.

Many technologies for proteome analysis are in use [21], [22]. In this study, we conducted a comparative proteomics analysis for four strains with different geospatial and temporal characteristics by performing 2-DE, and obtained their core and pan proteomes. We found that the proteome was highly conserved for the four *S*. Paratyphi A strains, consistent with the conservative genomes of *S*. Paratyphi A. However, some of the core proteins had significant differences in abundance among the strains, suggesting that there are variations in the protein expression in different strains, even though the strains have strict convergence in their genomes.

## Materials and Methods

### 1. Strains

Among the strains collected during the surveillance of typhoid and paratyphoid fever in China, and from the PFGE (*Xba*I) subtyping database, we selected the *S*. Paratyphi A strains from patients in 2-DE analysis: YN07077 (isolated in Yunnan province in 2007) and GZ9A05036 (isolated in Guizhou province in 2005), which have the predominant PFGE subtype, and ZJ98053 (isolated in Zhejiang province in 1998), with the nondominant subtype, for the 2-DE analysis. Strain ATCC 9150, which was isolated in Malaysia in 1993 clinically, was also included for comparison, it has a different PFGE subtype from the other three strains.

### 2. PFGE

We performed PFGE according to the method previously conducted in the paper [23].

### 3. Protein Extraction

The protein samples used for 2-DE were prepared according to the protocol described in a previous study [24]. In brief, the strains were cultured in Colombia blood agar for 16–18 hours, then the cells were scraped from four plates (9 cm in diameter) and washed four times in ice-cold low salt PBS. The cells were resuspended in deionized water and urea (7 M), thiourea (2 M), CHAPS (4%) and IPG buffer (1%), then DTT (1%) was added respectively, in a final volume of 5 ml. A protease inhibitor cocktail tablet (Roche applied science) was added to each sample. The samples were sonicated to lyse the cells, then 125 µg RNase A and 50 U DNase were added. The samples were kept at ambient temperature for 1 hour to make proteins sufficient dissolution, centrifuged at 40,000×g for 1 hour, then the supernatant was collected and the protein content was quantified with the PlusOne Quant Kit. The samples (800 µg protein) were aliquotted and either directly used for IEF or frozen at −80°C until use.

### 4. 2-DE and Image Scanning

Isoelectric focusing (IEF; 17 cm, pH 4–7, Bio-Rad; 18 cm, pH 6–11, Amersham Biosciences) and 12.5% sodium dodecyl sulfate polyacrylamide gel electrophoresis (SDS-PAGE) were performed according to the manufacturer’s instructions (Bio-Rad, PROTEAN IEF CELL, Protean II Xi apparatus). Briefly, passive rehydration was performed for 4 hours, and active rehydration was performed for 8 hours at 50 V, and IEF was conducted using the following conditions: 300 V linear for 1 hour, 600 V linear for 1 hour, 1000 V linear for 1 hour, 8000 V linear for 1 hour and 8000 V rapid for 8 hours. After the IEF and equilibration, the proteins were transferred by SDS-PAGE, using 10 mA for the electrophoresis of each strip for 30 minutes, which was then increased to 30 mA until the bromophenol blue line just shifted off of the lower edge of the gel. The procedure was then stopped, and the gel was dyed with Coomassie blue G-250. The gels were scanned with a UMAX2100XL device (Umax Technologies Inc.). All the samples were replicated the same procedure for three times.

### 5. In-gel Protein Digestion and Identification

The Coomassie-stained protein spots were cut and in-gel protein digestion was conducted as the previously described protocol [25]. Protein identification was carried out by using tandem matrix-assisted laser desorption/ionization time-of-flight (MALDI-TOF/TOF) mass spectrometry (MS, 4700 MALDI-TOF/TOF Mass Spectrometer, Applied Biosystems) as described previously [26]. The spectrum of every sample was acquired in the mass range between 800 and 4000 Da by using 1500 laser shots. MS/MS spectra were acquired by using 2000 laser shots with air as the collision gas. The single charged peaks were analyzed by using an interpretation method provided in the 4000 Series ExplorerTM software version 3.0, which selected the five most intense peaks and automatically generated the MS/MS spectra by excluding the peaks associated with the matrix and those were formed due to trypsin autolysis. The spectra were processed and analyzed by the Global Protein Server Workstation (GPS Applied Biosystems, Foster City, CA, USA), which uses internal Mascot v2.1 software for searching the peptide mass fingerprints. The searches were performed by using the NCBI non-redundant protein database (ftp://ftp.ncbi.nih.gov/blast/db/FAST/nr.gz, updated in 2011) with the following criteria: NCBI bacteria database; trypsin digestion; Moxidation and iodoacetamide alkylation as the variable modifications; missed digestion site of 1; and the MS mass error of 0.1 Da. Identifications with a GPS confidence interval greater than 95% were accepted. The inversion database was used to remove false positives (Protein identification was listed in Table S1 and Table S2, MS map of some proteins was listed in Attachement S2).

### 6. Data Analysis

An analysis of the proteomic data was performed using the PDQuest™ Advanced 2-DE Analysis software program. We used the basic model and default parameters (Attachement S1). After matching the spots using the software program, we revised the protein spot identification manually. Each spot displayed in all four gels was allocated to the core proteins, while spots displayed in only one strain were considered to be specific proteins. The data could be output using the following steps within the same window: File, Export, Export (Text) Experiment, Spot data by gel. We selected the center position option, so the (X, Y) values for each protein could be obtained. To normalize the coordinate values, all of the core proteins in each strain were designated to use the same coordinate value as ATCC9150, while the other shared proteins (minus the core proteins) were normalized using the same coordinate values as ATCC 9150, ZJ98053 or YN07077. For example, when a spot was found for ATCC 9150, its coordinate value in all strains that displayed the spot was designated to be the same as in ATCC 9150. Spots that were not present in ATCC 9150, but were found for ZJ98053, were designated to be the same as in ZJ98053. If spots were not found in either ATCC 9150 or ZJ98053, but were displayed in the gels for YN07077, its coordinate value would be designated to be the same as that in YN07077. Specific proteins for each strain were assigned an original coordinate value.

A scatter plot for pan proteins was generated using the Origin software program (Origin Lab), since each protein in each strain has a specific coordinate value (X, Y). Red represented the core proteins shared by all four strains, blue represented ATCC 9150-specific proteins, green represented ZJ98053-specific proteins, dark green represented YN07077-specific proteins, cyan represented GZ9A05036-specific proteins and black was used to indicate proteins other than the core and specific proteins.

Core protein and pan protein trend lines were generated using the Origin software program. A similarity matrix was generated according to the r values produced by the PDQuest software, version 8.0.1 (Bio-Rad).

Functional protein assignments were based on notation and classification on Tigr website (http://cmr.jcvi.org/tigr-scripts/CMR/CmrHomePage.cgi).

## Results

### 1. The Core Proteome and Pan Proteome of the Epidemic Strains

The 2-DE was performed within two pH ranges for the four strains of *S.* Paratyphi A, and the scanned patterns were analyzed using the PDQuest software program (Fig. S1–S8). Within the range of pH 4–7, 849, 858, 857 and 860 spots were detected in the strains ATCC 9150, ZJ98053, YN07077 and GZ9A05036 respectively, and 380, 389, 366 and 355 spots were detected within the range of pH 6–11 in these strains. Any spot detected in all four strains was considered to be a core protein, and the total number of core proteins identified was 739 and 318 within the ranges of pH 4–7 and pH 6–11. The core proteins covered from 85.9%–87.0% of the spots within the range of pH 4–7 and 81.8%–89.6% of the spots within the range of pH 6–11 in each strain, which suggested a high similarity in the protein expression among *S.* Paratyphi A strains, indicating that the proteome was highly conserved.

Within the ranges of pH 4–7 and pH 6–11, there were 946 and 435 pan proteins for the four strains. Core proteins covered a proportion of 78.1% and 73.1% of the pan proteins, confirming their conservation.

**Figure 1:**
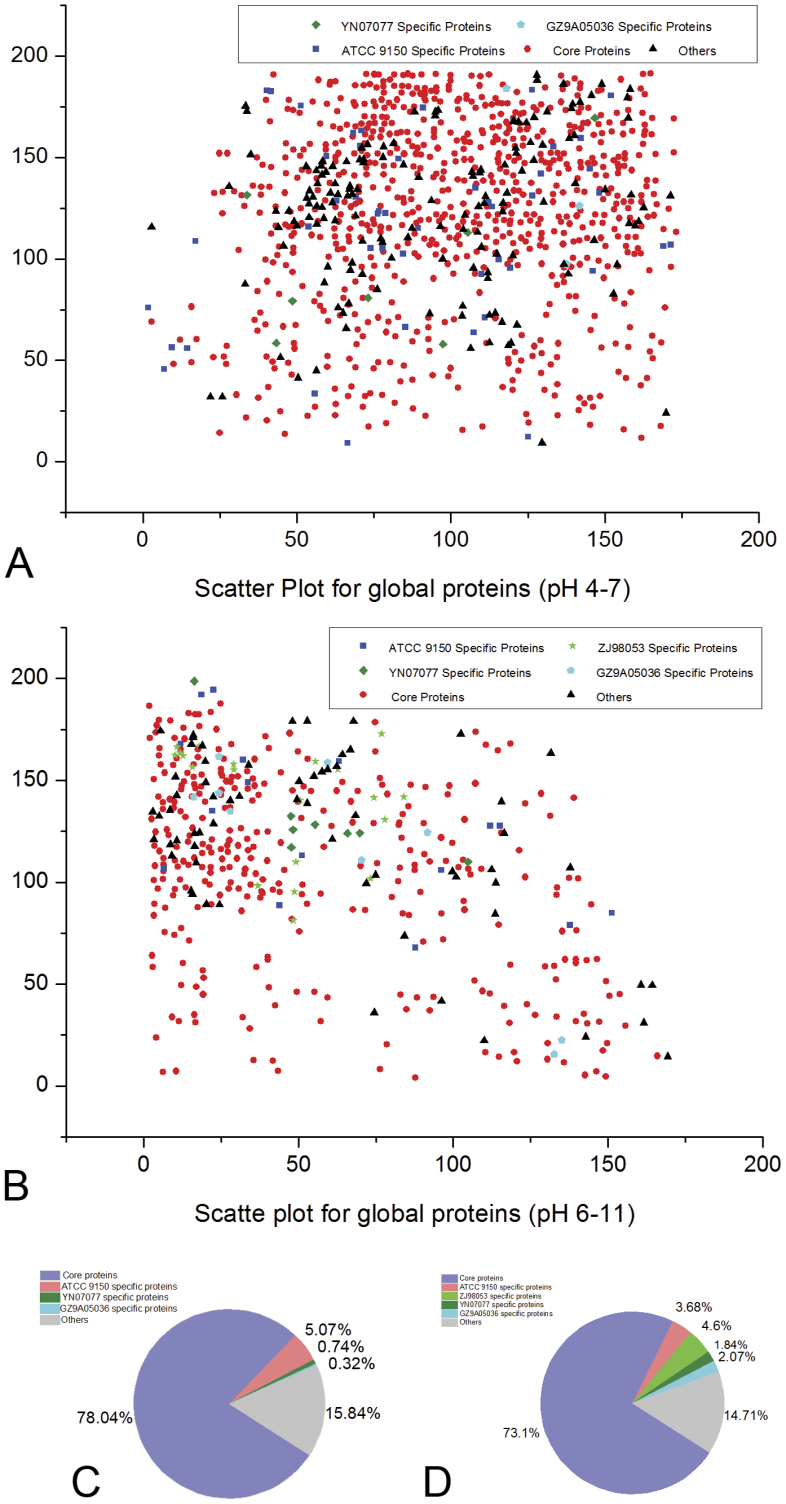
Scatter plot for the four strains of *S.* Paratyphi A pan proteins within pH range 4–7 and 6–11. A,B:Red spots represented core proteins, blue spots represented ATCC 9150 specific proteins, dark green spots represented YN07077 specific proteins, cyan spots represented GZ9A05036 specific proteins, black spots represented the other proteins except core or specific proteins in each strain; C,D represented proportion of the above proteins covered in the pan proteins within pH ranges 4–7 and 6–11 respectively.

To display the proportions of core proteins and strain-specific proteins in pan proteins, we drew scatter diagrams to show the pan proteome within the two pH ranges. The principle and process have already been described above. In brief, in the scatter diagrams, specific proteins in the four strains were represented by four different colors (there were no specific proteins for ZJ98053 within the pH range of 4–7). Core proteins are presented in red. The proteins other than the core proteins and specific proteins are shown in black (Fig. 1). We also presented a constitution map to show the proportion of core proteins and strain-specific proteins within the pan proteins.

**Figure 2:**
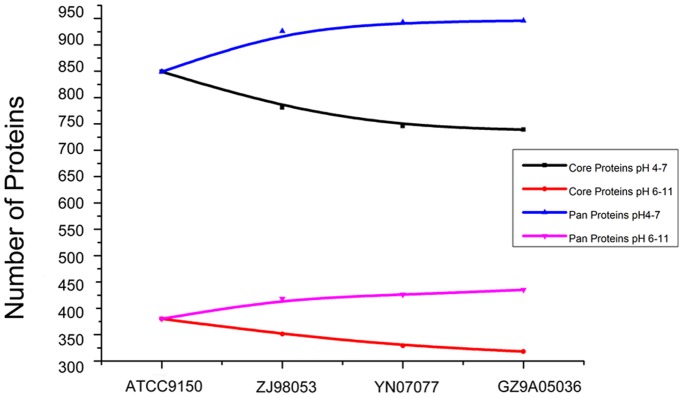
Number treadline for core and pan proteins when introducing more strains. Black and red lines showed core proteins tread lines within range pH–7 and pH 6–11; Blue and pink lines showed pan proteins tread lines within range pH –7 and pH 6–11.

The trend lines for the core and pan proteins exhibited the amount of protein change for each of the four *S*. Paratyphi A strains (Fig. 2). From strain ATCC 9150 to ZJ98053, which were isolated in 1993 and 1998, respectively, the number of pan proteins significantly increased. During this period, the incidence of paratyphoid fever increased dramatically in Southeast Asia and China. After adding strains YN07077 and ZJ98053, the slope of the increase slowed down, indicating that the proteome did not change very much. As far as the core protein trend line was concerned, it decreased quickly at the beginning and then slowed down, but the core proteins still covered a large proportion of the total proteins in each strain, suggesting that *S*. Paratyphi A has a conservative proteome.

The above data showed the expression level of proteins included in the core proteome, which include crucial proteins involved in the normal biological processes occurring within cells, which maintain the cells’ survival and basic physiological processes. The core proteome was distinguished from the core genome, because the latter is only theoretically crucial, and the gene transcription has not been confirmed.

**Figure 3:**
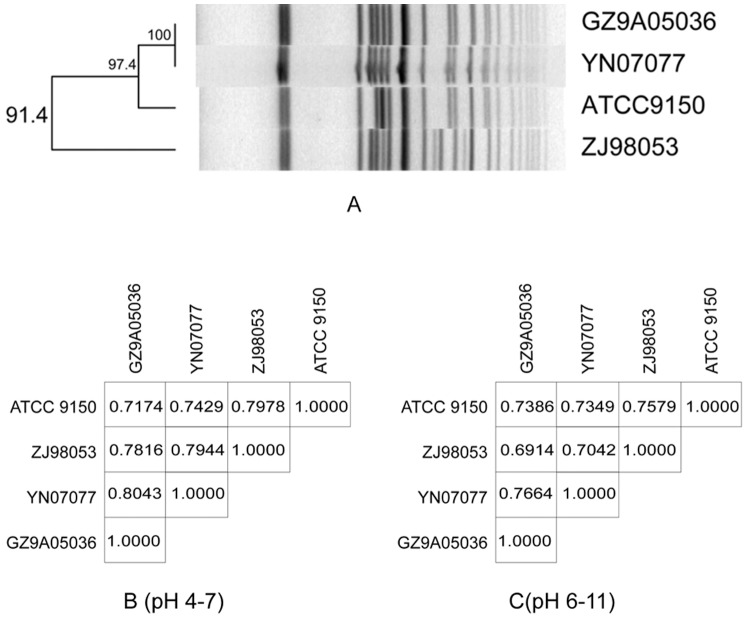
PFGE clustering and matrix for the four strains of *S.* Paratyphi A. A, Strain YN07077 and GZ9A05036 were the predominant PFGE type, and strain ATCC 9150 was subdominant PFGE type, strain ZJ98053 was the non dominant PFGE type; C,D was the proteome matrix.

The two pairing proteome comparisons among these four strains displayed various similarities, which were somewhat consistent with the PFGE clustering. However, there were also many differences among the strains (Fig. 3). Strains YN07077 and GZ9A05036 were the closest (with similarity of 80.4%) in terms of the protein pattern, and they had the same PFGE pattern. Compared to ATCC 9150, ZJ98053 was more similar to YN07077 and GZ9A05036 in terms of the proteome pattern, with similarity values of 79.44% and 78.16% respectively. Strain ATCC 9150 showed less similarity to YN07077 and GZ9A05036 (74.3% and 71.7%) than strain ZJ98053. Since strain ATCC 9150 was isolated in Malaysia in 1993, while strains ZJ98053, YN07077 and GZ9A05036 were from adjacent provinces in China, this suggests that the geospatial and temporal characteristics of the strains influence their proteomic pattern. In terms of the PFGE subtyping, strain ZJ98053 showed a nondominant pattern, ATCC 9150 showed a subdominant pattern and strains YN07077 and GZ9A05036 showed a predominant pattern. Strain ATCC 9150 was closer to YN07077 and GZ9A05036 than to ZJ98053 in terms of PFGE clustering. The differences in the proteomic and genomic patterns were likely due to the fact that the proteomic studies explored the more rapid proteomic response in cells when they were adapting to the environment around them, while the genome may take a longer time to show changes.

### 2. Constitution of the Expressed Proteins

**Figure 4:**
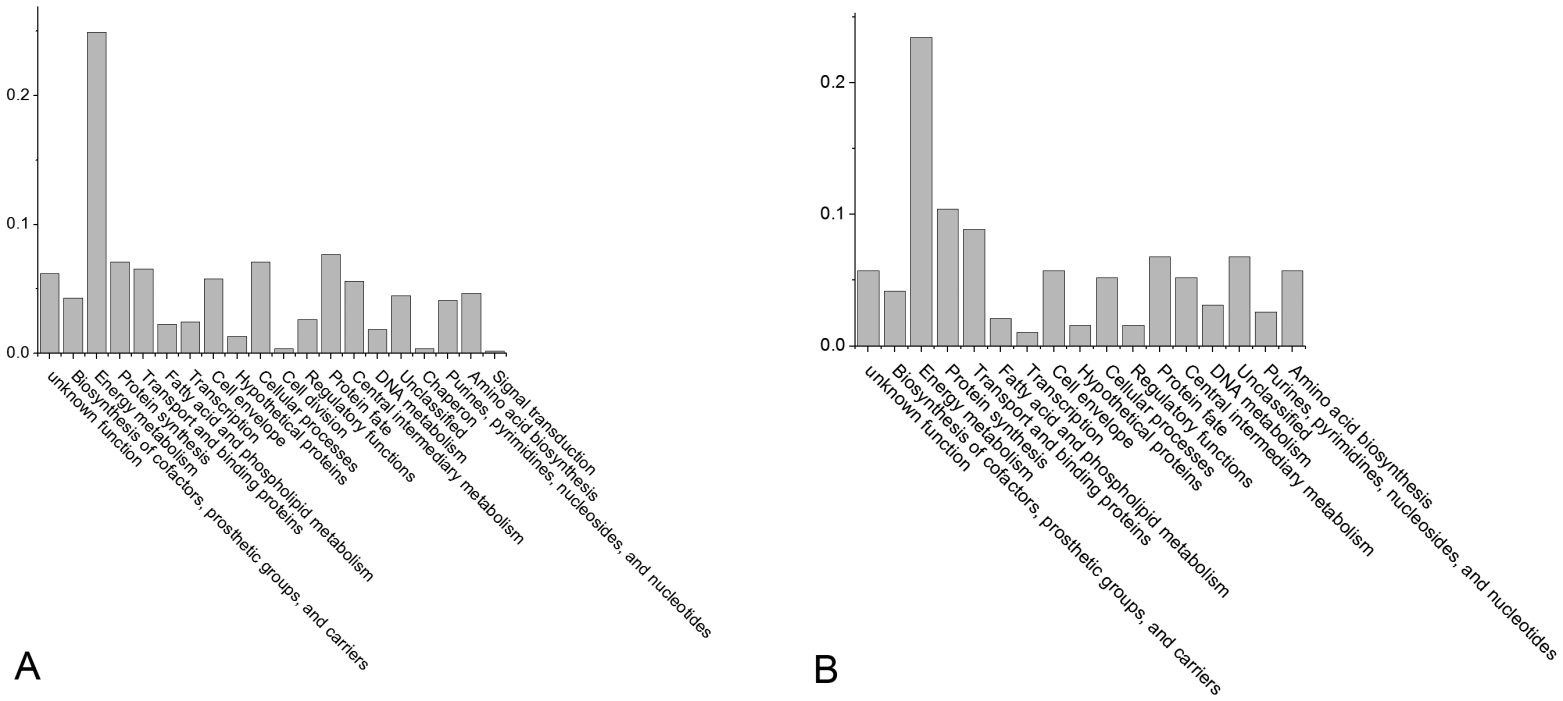
Functional categories of the core proteins within pH ranges 4–7 and 6–11. Each column represented the proportion of the protein number in this category to the total number of core proteins. A represents pH–7 and B represents pH 6–11.

**Figure 5:**
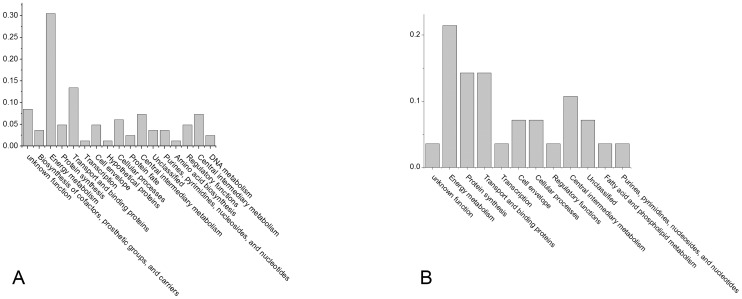
Functional categories of the non core proteins within pH range 4–7 and 6–11. Each column represented the proportion of the protein number in this category to the total number of non core proteins. A represents pH–7 and B represents pH 6–11.

Among the core proteins, the largest functional category was energy metabolism, then protein fate, protein synthesis, cellular processes, transport and binding proteins, central intermediary metabolism, etc. (Fig. 4). The functional constitution of the pan proteins other than the core proteins was slightly different from that of the core proteins. Energy metabolism was still the main category, but transport and binding proteins was the second most common functional category (Fig. 5).

### 3. Diverse Expression Levels of the Core Proteins

**Figure 6:**
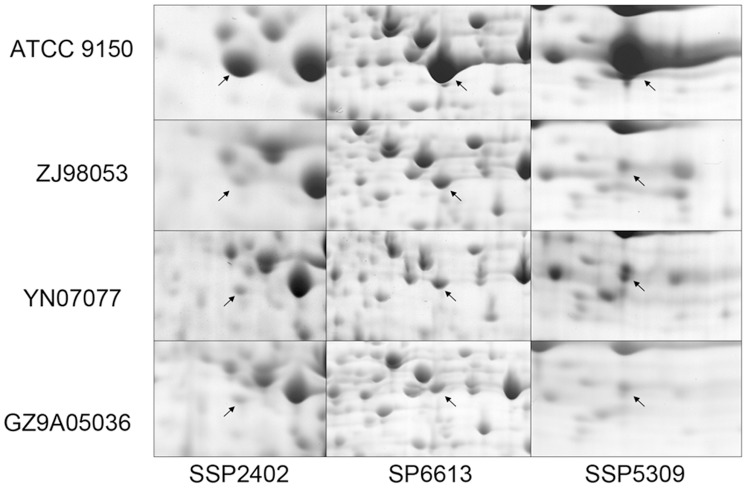
Spots with a higher protein expression level in ATCC 9150 than in the other *S*. Paratyphi A strains. The lines from the left to the right were the different expression level of core proteins SSP2402 (pH 4–7), SSP6613 (pH 4–7), SSP5309 (pH 6–11).

**Figure 7:**
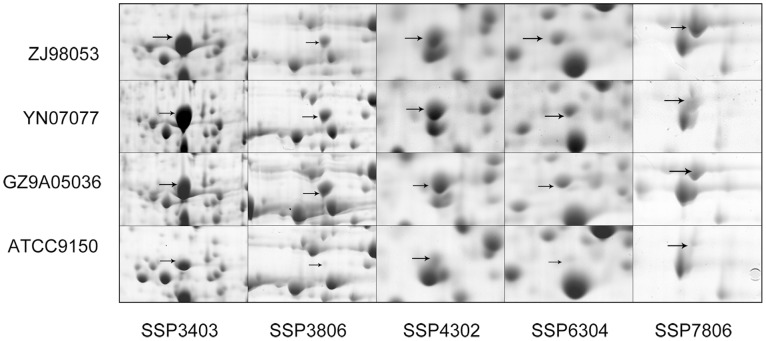
Spots with a lower protein expression level in ATCC 9150 than in the other *S*. Paratyphi A strains in pH 4–7.

Although these four *S*. Paratyphi A strains had a conserved proteome and they shared over 80% of their proteins, differences in the abundance of some protein spots were observed among the strains. Fig. 6 showed that some spots had a higher abundance in ATCC9150 than in the other three strains. Fig. 7 showed that other spots had a lower abundance in ATCC9150 than in the other three strains. Of these differentially-expressed spots, strain ZJ98053 had a more consistent protein expression level with YN07077 and GZ9A05036 compared to ATCC 9150, however, its proteome had a higher regression value with strain ATCC 9150 than with the other two strains (Fig. 3).

### 4. Strain-specific Proteins

We blasted all the coding genes for the MS identified proteins to ATCC9150 genome, but did not find new acquired gene products. All proteins were variants of the core proteins and non-core proteins.

## Discussion

In this study, the core proteome and pan proteome of four *S*. Paratyphi A strains cultured under laboratory conditions were compared, based on the core genome and pan genome comparison method. The previous genome comparisons revealed that *S*. Paratyphi A was highly clonal [10], [17]. We also found that there was limited genetic diversity in terms of the level of protein expression when strains were cultured under the same conditions. In the four tested strains, the core proteins covered a large proportion (>70%) of the pan proteomes. For each strain, the core proteins covered a proportion from 81.8% to 89.6% of the global proteins. Thus, the proteome of *S.* Paratyphi A was also highly conserved, which was consistent with the highly clonal genome.

The PFGE cluster analysis showed that strain YN07077 had the same pattern as GZ9A05036, less similarity with ATCC9150 and much less similarity with ZJ98053. Nevertheless, based on the regression matrix derived from the proteomic analysis, strain ZJ98053 was approaching YN07077 and GZ9A05036 in similarity, with less in common with ATCC9150. In terms of the amount of proteins, strains YN07077, GZ9A05036 and ZJ98053 had 813 (pH 4–7) core protein spots, which decreased to 739 (pH 4–7) after adding strain ATCC 9150, which indicated that similar genomes do not necessarily result in similar proteomes. Although the *S*. Paratyphi A strains had both conservative proteomes and genomes, they actively displayed distinct metabolic and other characteristics, which were not apparent at the genome level. Moreover, strains YN07077, GZ9A05036 and ZJ98053 were isolated from very close geographical regions, which might be the epidemiological basis for their high similarity in terms of the proteome, and their trend lines for core proteins and pan proteins exhibited no big changes and there were not significant differences between their proteomes, suggesting that the genomes and expression profiles of these strains were quite conservative, and that they had undergone stable evolution.

According to the functional classification of core proteins, the function of most of the core proteins was mainly focused on the survival of the organisms. Interestingly, some of the pan proteins (excluding the core proteins) fit in similar functional categories, which may reflect the high concordance of the expression profiles of these strains based on their conservative genomes. When the bacteria were grown under nutrient-rich conditions, the spread of the functional classification was nonspecific, because they were mainly experiencing routine metabolism that did not require new adaptations to improve survival.

Although *S*. Paratyphi A had a highly conserved proteome in terms of the protein species, some core proteins had significant fluctuations with regard to their abundance between strains. Strains YN07077, GZ9A05036 and ZJ98053 had some protein spots that were expressed at a similar abundance, such as spots SSP 3403, SSP 3806, SSP 4302, SSP 6304 and SSP 7806 at pH 4–7 and SSP 7117 at pH 6–11, which were expressed at a much higher abundance in these three strains than in the ATCC9150 strain. Both spots SSP 3403 and SSP 3806 were identified as outer membrane protein A, the surface-exposed porin proteins in high-copy number [27], which may play an important role in the structural stability and in the maintenance of the cell morphology, but has low-efficiency porin activity [28], [29], [30], [31]. It exposes to and interactes with outside circumstance factors. Their variants with subtle difference on modifications might adapt to diverse environments and host immunity, which might subsequently develop to inherited and characterized phenotypes. SSP 4302, SSP 7802 and SSP 7806 were correlated to the central stationary-phase-specific sigma subunit of RNA polymerase σ^s^ [32], [33], SSP 4302 (*arcA*) is a negative regulator for *rpoS* [34], SSP 7802 and SSP 7806 were positively regulated by *rpoS* [35]. It has been proved that *rpoS* was essential for *Salmonella* virulence, *rpoS* mutant of serovar Typhi is less cytotoxic for macrophages than the parental strain, therefore *rpoS* maybe involved in the virulence of serovar Typhi [36]. *S*. Paratyphi A has similar infection mechanism to *S*. Typhi, we could speculate that different growth status and cytotoxicity of bacteria might result in diverse expression of response factors in the regulative cascade.

However, some core proteins had a higher abundance in the ATCC9150 strain than in the other three strains, such as SSP 2402, SSP 5309 and SSP 6613. Genes of SSP 2402 (*rbsK*) and SSP 5309 (*rbsB*) locate in the same operon, which participate D-Ribose transportation and utilization [37]. This operon is transposable [38]. Up to now the real role of the higher expression is still unknown, but it may imply their biological roles in vary degrees in different strains and need further studies in detail.

ZJ98053 was in the middle in terms of its year of isolation (1998) compared with the other three strains (1993 for ATCC9150, 2005 for GZ9A05036 and 2007 for YN07077), but it was geographically close to strains GZ9A05036 and YN07077, and it exhibited high genomic similarity to ATCC9150 and high proteomic similarity to YN07077 and GZ9A05036. It also showed independent characteristics from all the other strains. For example, spots SSP 3204, SSP 6703 and SSP 1405 were more abundant in strain ZJ98053 than in the other three strains, which suggests that ZJ98053 might have evolved separately from the other three strains.

The above differentially-expressed core protein spots were spread throughout various metabolic pathways. The variable expression levels of core proteins revealed the metabolic diversity present in the different strains. Thus, even core proteins produced under the same culture conditions can display diverse expression levels and different modifications to exert different functions, which eventually become a characteristic genetic phenotype [39], [40]. Such phenotypes were common in this study, and may have been connected to the function of the individual proteins.

With regard to the specific spots, we blasted (http://blast.ncbi.nlm.nih.gov/Blast.cgi) the gene sequence for the ATCC 9150 genome, and found that there were limited differences caused by the differences in the genome or pseudogenes. Most differentially-expressed spots were considered to have been caused by differences in the transcription level or post-translational modifications.

A high-throughput genome comparison can provide a detailed gene map, including the genes, their arrangement, recombination, pseudogene accumulation and similarity between strains, whereas information about the gene expression, protein modification and regulatory network cannot be obtained from such studies. Different expression profiles (including the protein species and their abundance) can be observed even when strains have the same or similar gene clusters, since large differences can arise due to differences in the gene expression, regulatory networks and protein modifications. Thus, biological studies, and interpreting the results of such studies, remain challenging even when the whole genome sequences are known. Proteomic studies can provide information about the true expression of the genes under the studied culture condition, and the core proteome reveals the conservative expression of the genomes of different strains under this condition. Further, proteomic comparisons may show the genome-based differences, and even evolutionary relationships, among the strains, even when the genome sequences are unknown.

In summary, we herein compared the core proteome and pan proteome of *S*. Paratyphi A strains isolated during recent epidemics. Our results may provide a new approach to analyzing the expression profiles of strains at the species level, which can help to understand their genetic differences, without requiring the genomic sequence, and can facilitate understanding their common biological processes under specific conditions, which will provide information about their fundamental metabolism and survival strategies. In addition, more sensitive and high-throughput technology, such as iTRAQ-based LC-MS/MS analyses, may make it possible to perform large scale analyses of proteomic data, and may also provide information for a powerful database that can be used to assess newly-identified or emerging strains.

## Supporting Information

Figure S1 **Two-dimensional electrophoresis and identified spots of whole-cell proteins for ATCC 9150 within pH range 4–7.**
(TIF)

Figure S2 **Two-dimensional electrophoresis and identified spots of whole-cell proteins for ATCC 9150 within pH range 6–11.**
(TIF)

Figure S3 **Two-dimensional electrophoresis and identified spots of whole-cell proteins for ZJ98053 within pH range 4–7.**
(TIF)

Figure S4 **Two-dimensional electrophoresis and identified spots of whole-cell proteins for ZJ98053 within pH range 6–11.**
(TIF)

Figure S5 Two-dimensional electrophoresis and identified spots of whole-cell proteins for YN07077 within pH range 4–7.
(TIF)

Figure S6 **Two-dimensional electrophoresis and identified spots of whole-cell proteins for YN07077 within pH range 6–11.**
(TIF)

Figure S7 **Two-dimensional electrophoresis and identified spots of whole-cell proteins for GZ9A05036 within pH range 4–7.**
(TIF)

Figure S8 **Two-dimensional electrophoresis and identified spots of whole-cell proteins for GZ9A05036 within pH range 6–11.**
(TIF)

Table S1 **Protein identification for the global spots of strain ATCC9150 and differential spots of strain ZJ98053, YN07077 and GZ9AO5036 within pH range 4–7.**
(RAR)

Table S2 **Protein identification for the global spots of strain ATCC9150 and differential spots of strain ZJ98053, YN07077 and GZ9AO5036 within pH range 6–11.**
(RAR)

Attachment S1 **Spots counting parameter for 2-DE map using PDQuest software.**
(SDX)

Attachment S2 **Mass spectrum identification for some proteins of** ***S*** **. Paratyphi A.**
(RAR)

## References

[1] Crump JA, Luby SP, Mintz ED (2004) The global burden of typhoid fever. Bull World Health Organ 82: 346–353. 15298225 PMC2622843

[2] Crump JA, Mintz ED (2010) Global trends in typhoid and paratyphoid Fever. Clin Infect Dis 50: 241–246. 20014951 10.1086/649541 PMC2798017

[3] Ochiai RL, Wang X, von Seidlein L, Yang J, Bhutta ZA, et al (2005) Salmonella paratyphi A rates, Asia. Emerg Infect Dis 11: 1764–1766. 16318734 10.3201/eid1111.050168 PMC3367370

[4] Dong BQ, Yang J, Wang XY, Gong J, von Seidlein L, et al (2010) Trends and disease burden of enteric fever in Guangxi province, China, 1994–2004. Bull World Health Organ 88: 689–696. 20865074 10.2471/BLT.09.069310 PMC2930361

[5] Yan M, Liang W, Li W, Kan B (2005) Epidemics of Typhoid and Paratyphoid Fever From 1995 Through 2004 in China. DISEASE SURVEILLANCE 20: 401–403.

[6] Sood S, Kapil A, Dash N, Das BK, Goel V, et al (1999) Paratyphoid fever in India: An emerging problem. Emerg Infect Dis 5: 483–484. 10341194 10.3201/eid0503.990329 PMC2640769

[7] Liang W, Zhao Y, Chen C, Cui X, Yu J, et al (2012) Pan-genomic analysis provides insights into the genomic variation and evolution of Salmonella Paratyphi A. PLoS One. 7: e45346. 10.1371/journal.pone.0045346 PMC3446902 23028950

[8] Parkhill J, Dougan G, James KD, Thomson NR, Pickard D, et al (2001) Complete genome sequence of a multiple drug resistant Salmonella enterica serovar Typhi CT18. Nature 413: 848–852. 11677608 10.1038/35101607

[9] Deng W, Liou SR, Plunkett G 3rd, Mayhew GF, Rose DJ, et al (2003) Comparative genomics of Salmonella enterica serovar Typhi strains Ty2 and CT18. J Bacteriol 185: 2330–2337. 12644504 10.1128/JB.185.7.2330-2337.2003 PMC151493

[10] McClelland M, Sanderson KE, Clifton SW, Latreille P, Porwollik S, et al (2004) Comparison of genome degradation in Paratyphi A and Typhi, human-restricted serovars of Salmonella enterica that cause typhoid. Nat Genet 36: 1268–1274. 15531882 10.1038/ng1470

[11] Kidgell C, Reichard U, Wain J, Linz B, Torpdahl M, et al (2002) Salmonella typhi, the causative agent of typhoid fever, is approximately 50,000 years old. Infect Genet Evol 2: 39–45. 12797999 10.1016/s1567-1348(02)00089-8

[12] Chen C, Zhao Y, Han H, Pang B, Zhang J, et al (2012) Optimization of pulsed-field gel electrophoresis protocols for Salmonella Paratyphi A subtyping. Foodborne Pathog Dis 9: 325–330. 22443482 10.1089/fpd.2011.1023

[13] Holt KE, Parkhill J, Mazzoni CJ, Roumagnac P, Weill FX, et al (2008) High-throughput sequencing provides insights into genome variation and evolution in Salmonella Typhi. Nat Genet 40: 987–993. 18660809 10.1038/ng.195 PMC2652037

[14] Baker S, Holt K, van de Vosse E, Roumagnac P, Whitehead S, et al (2008) High-throughput genotyping of Salmonella enterica serovar Typhi allowing geographical assignment of haplotypes and pathotypes within an urban District of Jakarta, Indonesia. J Clin Microbiol 46: 1741–1746. 18322069 10.1128/JCM.02249-07 PMC2395080

[15] Holt KE, Baker S, Dongol S, Basnyat B, Adhikari N, et al (2010) High-throughput bacterial SNP typing identifies distinct clusters of Salmonella Typhi causing typhoid in Nepalese children. BMC Infect Dis 10: 144. 20509974 10.1186/1471-2334-10-144 PMC2897797

[16] Holt KE, Dolecek C, Chau TT, Duy PT, La TT, et al (2011) Temporal fluctuation of multidrug resistant salmonella typhi haplotypes in the mekong river delta region of Vietnam. PLoS Negl Trop Dis 5: e929. 21245916 10.1371/journal.pntd.0000929 PMC3014949

[17] Baker S, Holt KE, Clements AC, Karkey A, Arjyal A, et al (2011) Combined high-resolution genotyping and geospatial analysis reveals modes of endemic urban typhoid fever transmission. Open Biol 1: 110008. 22645647 10.1098/rsob.110008 PMC3352080

[18] Jacobsen A, Hendriksen RS, Aaresturp FM, Ussery DW, Friis C (2011) The Salmonella enterica pan-genome. Microb Ecol 62: 487–504. 21643699 10.1007/s00248-011-9880-1 PMC3175032

[19] Pandey A, Mann M (2000) Proteomics to study genes and genomes. Nature 405: 837–846. 10866210 10.1038/35015709

[20] Naaby-Hansen S, Waterfield MD, Cramer R (2001) Proteomics–post-genomic cartography to understand gene function. Trends Pharmacol Sci 22: 376–384. 11431033 10.1016/s0165-6147(00)01663-1

[21] Encheva V, Wait R, Begum S, Gharbia SE, Shah HN (2007) Protein expression diversity amongst serovars of Salmonella enterica. Microbiology 153: 4183–4193. 18048932 10.1099/mic.0.2007/010140-0

[22] Steel LF, Haab BB, Hanash SM (2005) Methods of comparative proteomic profiling for disease diagnostics. J Chromatogr B Analyt Technol Biomed Life Sci 815: 275–284. 10.1016/j.jchromb.2004.10.072 15652816

[23] Ribot EM, Fair MA, Gautom R, Cameron DN, Hunter SB, et al (2006) Standardization of pulsed-field gel electrophoresis protocols for the subtyping of Escherichia coli O157:H7, Salmonella, and Shigella for PulseNet. Foodborne Pathog Dis 3: 59–67. 16602980 10.1089/fpd.2006.3.59

[24] Yuan J, Zhu L, Liu X, Li T, Zhang Y, et al (2006) A proteome reference map and proteomic analysis of Bifidobacterium longum NCC2705. Mol Cell Proteomics 5: 1105–1118. 16549425 10.1074/mcp.M500410-MCP200

[25] Ying T, Wang H, Li M, Wang J, Shi Z, et al (2005) Immunoproteomics of outer membrane proteins and extracellular proteins of Shigella flexneri 2a 2457T. Proteomics 5: 4777–4793. 16281178 10.1002/pmic.200401326

[26] Zhang MJ, Zhao F, Xiao D, Gu YX, Meng FL, et al (2009) Comparative proteomic analysis of passaged Helicobacter pylori. J Basic Microbiol 49: 482–490. 19455517 10.1002/jobm.200800372

[27] Confer AW, Ayalew S (2013) The OmpA family of proteins: roles in bacterial pathogenesis and immunity. Vet Microbiol 163: 207–222. 22986056 10.1016/j.vetmic.2012.08.019

[28] Jap BK, Walian PJ (1990) Biophysics of the structure and function of porins. Q Rev Biophys 23: 367–403. 2178269 10.1017/s003358350000559x

[29] Singh SP, Williams YU, Miller S, Nikaido H (2003) The C-terminal domain of Salmonella enterica serovar typhimurium OmpA is an immunodominant antigen in mice but appears to be only partially exposed on the bacterial cell surface. Infect Immun 71: 3937–3946. 12819080 10.1128/IAI.71.7.3937-3946.2003 PMC161966

[30] Sugawara E, Nikaido H (1992) Pore-forming activity of OmpA protein of Escherichia coli. J Biol Chem 267: 2507–2511. 1370823

[31] Sugawara E, Nikaido H (1994) OmpA protein of Escherichia coli outer membrane occurs in open and closed channel forms. J Biol Chem 269: 17981–17987. 7517935

[32] O’Neal CR, Gabriel WM, Turk AK, Libby SJ, Fang FC, et al (1994) RpoS is necessary for both the positive and negative regulation of starvation survival genes during phosphate, carbon, and nitrogen starvation in Salmonella typhimurium. J Bacteriol 176: 4610–4616. 8045891 10.1128/jb.176.15.4610-4616.1994 PMC196281

[33] Talukder AA, Yanai S, Nitta T, Kato A, Yamada M (1996) RpoS-dependent regulation of genes expressed at late stationary phase in Escherichia coli. FEBS Lett 386: 177–180. 8647276 10.1016/0014-5793(96)00426-7

[34] Sevcik M, Sebkova A, Volf J, Rychlik I (2001) Transcription of arcA and rpoS during growth of Salmonella typhimurium under aerobic and microaerobic conditions. Microbiology 147: 701–708. 11238977 10.1099/00221287-147-3-701

[35] Ibanez-Ruiz M, Robbe-Saule V, Hermant D, Labrude S, Norel F (2000) Identification of RpoS (sigma(S))-regulated genes in Salmonella enterica serovar typhimurium. J Bacteriol 182: 5749–5756. 11004173 10.1128/jb.182.20.5749-5756.2000 PMC94696

[36] Khan AQ, Zhao L, Hirose K, Miyake M, Li T, et al (1998) Salmonella typhi rpoS mutant is less cytotoxic than the parent strain but survives inside resting THP-1 macrophages. FEMS Microbiol Lett 161: 201–208. 9561749 10.1111/j.1574-6968.1998.tb12949.x

[37] Iida A, Harayama S, Iino T, Hazelbauer GL (1984) Molecular cloning and characterization of genes required for ribose transport and utilization in Escherichia coli K-12. J Bacteriol 158: 674–682. 6327617 10.1128/jb.158.2.674-682.1984 PMC215482

[38] Abou-Sabe M, Pilla J, Hazuda D, Ninfa A (1982) Evolution of the D-ribose operon on Escherichia coli B/r. J Bacteriol 150: 762–769. 6279571 10.1128/jb.150.2.762-769.1982 PMC216427

[39] Meysman P, Sanchez-Rodriguez A, Fu Q, Marchal K, Engelen K (2013) Expression divergence between Escherichia coli and Salmonella enterica serovar Typhimurium reflects their lifestyles. Mol Biol Evol 30: 1302–1314. 23427276 10.1093/molbev/mst029 PMC3649669

[40] Leekitcharoenphon P, Lukjancenko O, Friis C, Aarestrup FM, Ussery DW (2012) Genomic variation in Salmonella enterica core genes for epidemiological typing. BMC Genomics 13: 88. 22409488 10.1186/1471-2164-13-88 PMC3359268

